# Oxytocin Attenuates Expression, but Not Acquisition, of Sucrose Conditioned Place Preference in Rats

**DOI:** 10.3389/fnbeh.2020.603232

**Published:** 2020-12-15

**Authors:** Devon Patel, Megana Sundar, Eva Lorenz, Kah-Chung Leong

**Affiliations:** Department of Psychology, Trinity University, San Antonio, TX, United States

**Keywords:** oxytocin, sucrose, sugar, reward, conditioned place preference (CPP)

## Abstract

Maladaptation of reward processing for natural rewards, such as sucrose or sugar, may play a role in the development of diseases such as obesity and diabetes. Furthermore, uncovering mechanisms to disrupt or reverse maladaptation of reward-seeking behaviors for natural reinforcers can provide insight into treatment of such diseases, as well as disorders such as addiction. As such, studying the effects of potential pharmacotherapeutics on maladaptive sugar-seeking behavior offers valuable clinical significance. Sucrose conditioned place preference (CPP) paradigms can offer insight into aspects of reward processes as it provides a way to assess acquisition and expression of context-reward associations. The present study examined the effect of peripheral oxytocin injections on sucrose CPP in rats. Oxytocin, when administered prior to CPP test, attenuated expression of sucrose CPP. However, oxytocin, when administered during sucrose conditioning, did not affect subsequent place preference. These findings suggest oxytocin sufficiently attenuates expression of sucrose-associated place preference.

## Introduction

Overconsumption of sugar has been shown to lead to a myriad of diseases. Excess sugar can lead to an increased risk of developing cardiovascular disease and type 2 diabetes (Stanhope, 2016). This excessive consumption also promotes obesity and increases visceral fat volume, which is associated with risk factors for metabolic disease (Carr et al., 2004). The fact that a wide variety of foods contain added sugar perpetuates the issue. For example, sugar-sweetened beverages, such as fruit juice and soda, account for the primary source of added sugar in the diets of people over the age of 2-years-old in the United States (Bailey et al., 2018). While numerous studies have examined potential therapeutic targets for the treatment of drug-related behaviors and addiction, relatively few studies have examined therapeutic targets that modulate reward behaviors related to natural reinforcers. The present study aimed to examine the effect of a potential therapeutic target, oxytocin, on sucrose-associated place preference behavior.

Oxytocin is a neuropeptide implicated in a variety of behaviors including addictive processes, stress responses, and social affiliations (Lee et al., 2016). Previous experiments have shown that oxytocin affects the seeking and reinstatement behavior to a variety of drugs of abuse, including alcohol (King and Becker, 2019), methamphetamine (Carson et al., 2010), and cocaine (Morales-Rivera et al., 2014; Leong et al., 2016). Early evidence has also demonstrated that oxytocin reduced reinstated sucrose-seeking behavior (Zhou et al., 2015a). Additionally, studies have demonstrated that oxytocin attenuated sucrose-seeking behavior and taking behavior in a sex-dependent manner (Cox et al., 2013). Here, we examine whether oxytocin is also effective in disrupting the acquisition and expression of a sucrose-associated place preference.

The conditioned place preference (CPP) paradigm allows experimenters to study context-dependent reward memory and the rewarding value of natural and drug stimuli (Carr et al., 1989; Figlewicz et al., 2001). The paradigm provides an effective method for examining reward processes without necessarily the formation of substantial addictive behaviors while still providing insight into the processes that might precede it. The CPP paradigm has commonly been employed to investigate drug reinforcers, including cocaine (Nomikos and Spyraki, 1988) and methamphetamine (Tuazon et al., 1992). Depending on the reinforcer, CPP can be achieved with a single trial (morphine: Mucha et al., 1982; cocaine: Bardo et al., 1986; methamphetamine: Baracz et al., 2012), or can also be induced after multiple administrations (Nomikos and Spyraki, 1988). Recently, a growing body of literature has demonstrated that CPP is a valuable paradigm to study natural reinforcers as well (Bardo and Bevins, 2000). The CPP paradigm is ideal for investigating the effects of oxytocin administration on the processes related to sucrose reward-related behavior.

The goal of the present study is to evaluate the role of oxytocin in attenuating sucrose-associated place preference through the establishment of sucrose CPP. Although high sucrose consumption is a prevalent cause for many health conditions, pharmacological interventions of sucrose-seeking behavior have not been thoroughly elucidated. Specifically we demonstrate here that administration of peripheral oxytocin prior to expression, but not during conditioning, of sucrose place preference effectively reduces sucrose-associated place preference behavior, indicating that oxytocin is an effective pharmacological option to disrupt sucrose-associated reward behaviors. Potential insights into the use of oxytocin as an option to disrupt maladaptation of reward processes are also discussed.

## Methods

### Subjects

Adult male (maintained at 275–300 g throughout study) Sprague-Dawley rats (Charles River Laboratories, *N* = 35) were used in this study. Rats were single-housed on a reverse 12:12 light-dark cycle in a set temperature and humidity-controlled vivarium. During the experiment, animals were food-restricted to 10 g of chow daily and water *ad-libitum*. All procedures were approved by the Institutional Animal Care and Use Committee (IACUC) of Trinity University.

### Apparatus

The CPP apparatus (Panlab–Harvard Apparatus) was composed of two Plexiglas compartments (each: 30.0 cm length × 30.0 cm width × 34.0 cm height) that were connected by a central corridor (10.0 cm length × 8.0 cm width × 34.0 cm height). One compartment had a black floor and walls, while the other compartment had a white floor and walls. The central corridor had gray walls and a gray floor. All floor and wall textures were consistent. The animal's location and transitions between compartments were measured using pressure plates under the floors of the gray and black compartments and the data were relayed to the tracking software, PPCWIN, via a control panel (Panlab–Harvard Apparatus). The doors in between the compartments were manually operated sliding doors.

### Drugs

Oxytocin (Cell Sciences) was dissolved in 0.9% NaCl saline and administered intraperitoneally (i.p.) at a dose of 1 mg/kg.

### Behavioral Protocol

#### Sucrose Priming

For 2 days prior to the start of the habituation phase, all rats were food restricted to 10 grams of standard chow and given 10 sucrose pellets (45 mg each, Bio-Serv) each, daily.

#### Habituation

Behavioral training began with a habituation trial to determine baseline place preference. No sucrose was provided for the duration of habituation. During habituation, all rats were placed in the gray central corridor. Then, the doors were opened so the animal was allowed free access to both of the compartments for an entire 15-min session. During the session, the amount of time the rat stayed in each compartment was measured and the percentage of time spent within either compartment was calculated to determine the animal's baseline preference. Whichever compartment that the rat did not display greater baseline preference for was designated as the sucrose-paired compartment during subsequent conditioning trials.

#### Conditioning

Rats were randomly assigned to the different treatment groups and counterbalanced such that all the rats did not start conditioning in the same compartment each day (i.e., subsequent sessions started in opposite chambers). Animals received three sucrose-paired sessions in the non-preferred compartment on alternate days (e.g., Days 1, 3, 5) and three unpaired sessions on the days in between sucrose-paired sessions. Training sessions ran for 6 consecutive days and lasted 30-min each. During the sucrose conditioning days, animals initially received five sucrose pellets followed by five more every 10 min (15 pellets total), delivered at regular intervals by the experimenter. Sucrose pellets were consumed in all conditions. On unpaired sessions rats received “sham deliveries,” in which the experimenter would make identical movements every 10 min without the actual delivery of sucrose pellets.

#### Testing

On the 7th day (Test Day), animals underwent the same procedure as in habituation to determine if sucrose conditioned place preference had been established. No sucrose was provided for the duration of testing. Depending on the experimental group, the animals either received an injection of saline (VEH) or oxytocin (OXY) test day (described below).

### Experimental Procedures

#### Pre-test Oxytocin (Exp. 1a)

**Figure 1:**
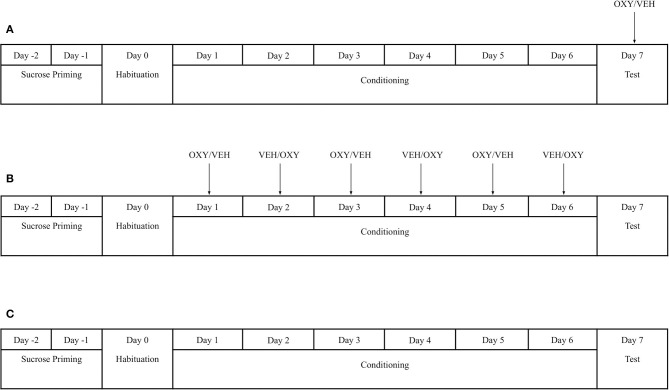
Experimental timeline and pharmacological manipulation in **(A)** Experiment 1a **(B)** Experiment 1b **(C)** Experiment 1c. Sucrose-conditioning sessions were counterbalanced in Experiment 1a and 1b. In Experiment 1b, animals in the OXY treatment group received OXY injections only prior to sucrose-paired conditioning sessions. Animals in Experiment 1c received no access to sucrose pellets during conditioning or any injections of OXY or VEH. VEH = Vehicle; OXY = Oxytocin.

To determine the effect of oxytocin on the expression of sucrose-associated place preference, all rats underwent sucrose priming, habituation, conditioning, and testing as adapted from Figlewicz et al. (2001) and described above (Figure 1). On day 7 (Test Day), rats were either injected with OXY (1 mg/kg; i.p.; *n* = 7) or VEH (*n* = 7) 35 min prior to testing. The test session was identical to the habituation procedure.

#### Oxytocin During Conditioning (Exp. 1b)

To determine the effect of oxytocin on the acquisition of sucrose-associated place preference, all rats underwent identical behavioral procedures as Exp. 1a. Rats either received an injection of OXY (1 mg/kg; i.p.; *n* = 8) or VEH (*n* = 6) 35 min prior to the start of sucrose-paired sessions during conditioning. All rats also received an injection of VEH 35 min prior to the start of non-sucrose-paired sessions. No injections were given on test day.

#### No Manipulation (Exp. 1c)

To determine that development of any place preference was specifically due to sucrose-associated conditioning, one group of rats (*n* = 7) did not receive sucrose pellets during conditioning. The animals received sucrose pellets only during priming but did not receive any sucrose pellets during behavioral training or test. All rats underwent the same conditioning and testing procedures without drug manipulation.

### Statistical Analyses

Amount of time and percentage of time spent in chambers was calculated using PPCWIN software. Percentage of time spent in the sucrose-paired chamber was calculated as time spent in sucrose-paired chamber/total time spent in black and white chambers. Time spent in the gray chamber was omitted as no time was spent in this chamber during conditioning trials. Two-way repeated measures ANOVAs were performed to determine between-group differences in place preference between OXY and VEH treated animals and within-group differences between baseline and test preference. *Post-hoc* Sidak's multiple comparisons test were performed to compare differences in time spent in sucrose-paired chambers within habituation or test across treatment groups. All data are presented as the mean ± S.E.M. and α was set at *p* < 0.05.

## Results

### Pre-test Oxytocin Disrupts Expression of Sucrose Conditioned Place Preference

**Figure 2:**
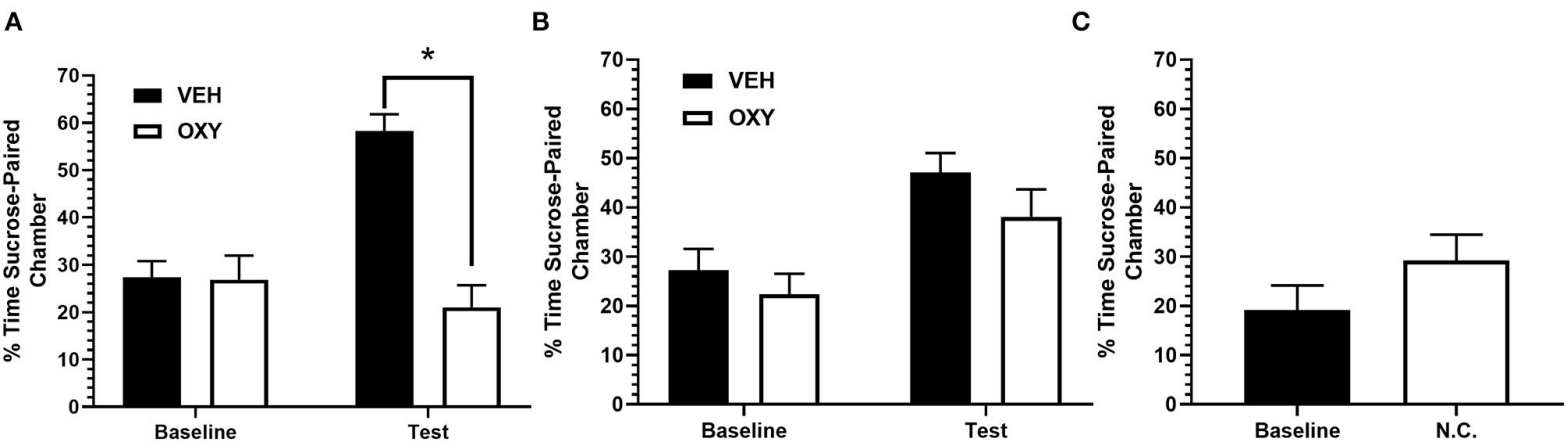
Percentage of time spent in sucrose-paired chamber on test. **(A)** Rats injected with OXY displayed an attenuation in time spent in the sucrose-paired chamber during preference test relative to VEH treated rats. There was no difference in baseline preference between VEH and OXY treated rats. **(B)** All rats successfully acquired sucrose-associated conditioned place preference. There was no difference in time spent in sucrose-paired chamber during preference test between OXY or VEH administered during conditioning. **(C)** Non-conditioned rats, receiving no sucrose-pairings or drug manipulation, spent displayed no difference in time spent in sucrose-paired chamber relative to baseline. VEH = Vehicle; OXY = Oxytocin; N.C. = No conditioning * indicates significant difference at *p* < 0.05.

A two-way repeated measures ANOVA revealed a significant main effect of treatment group [*F*_(1, 12)_ = 20.94, *p* < 0.01] and significant main effect of test [*F*_(1, 12)_ = 8.059, *p* < 0.05]. There was a statistically significant interaction between the effect of treatment and test on sucrose preference [*F*_(1, 12)_ = 17.34, *p* < 0.01]. A *post-hoc* Sidak's multiple comparisons test revealed a significant difference in time spent in the sucrose-paired chamber during test between OXY (*M* = 21.01, *SD* = 12.35) and VEH (*M* = 58.22, *SD* = 9.54; *p* < 0.01) treated animals but no significant difference between both groups in baseline preference (OXY: *M* = 26.85, *SD* = 12.35; VEH: *M* = 27.33, *SD* = 9.12; n.s.). These results suggest that OXY treated animals displayed attenuated expression of sucrose-associated place preference relative to VEH treated animals at test (Figure 2A).

### Oxytocin During Conditioning Did Not Affect Acquisition of Sucrose Place Preference

Animals in Exp. 1b underwent similar behavioral procedures as Exp. 1a except OXY was administered prior to conditioning trials instead of prior to test. A two-way repeated measures ANOVA revealed a significant main effect of test [*F*_(1, 12)_ = 18.14, *p* < 0.01]. There was no significant main effect of treatment group [*F*_(1, 12)_ = 1.730, n.s.] and no significant interaction [*F*_(1, 12)_ = 0.2431, n.s.]. A *post-hoc* Sidak's multiple comparisons test revealed no significant difference in time spent in the sucrose chamber between treatment groups at test (OXY: *M* = 38.11, *SD* = 15.64; VEH: *M* = 47.08, *SD* = 9.67; n.s.) or baseline (OXY: *M* = 22.38, *SD* = 11.72; VEH: *M* = 27.23, *SD* = 10.63, n.s.), suggesting that OXY administration during conditioning did not affect acquisition of sucrose-associated place preference relative to VEH treated animals (Figure 2B).

### Animals Showed No Change in Baseline Behavior Without Sucrose Conditioning

In order to determine that place preference behavior was a result of sucrose-pairing during conditioning, rats underwent the same procedure as Exp. 1a and 1b without any sucrose-pairings. A paired *t*-test revealed no significant difference in time spent in the sucrose-paired chamber during test (*M* = 29.23, *SD* = 13.79) and baseline preference [*M* = 19.15, *SD* = 13.16; *t* (6) = 1.78, n.s.; Figure 2C] when animals were not exposed to sucrose-pairings in either chamber. These results suggest that the place preference developed in prior experiments were a result of sucrose-pairings within those chambers.

## Discussion

The present study demonstrates that sucrose conditioned place preference was successfully acquired in animals that received sucrose-pairings in a specific context and that peripheral administration of oxytocin significantly disrupted expression of conditioned place preference when administered before the test. However, oxytocin administered during conditioning trials did not affect the acquisition of sucrose conditioned place preference. These findings suggest that oxytocin sufficiently attenuates expression of sucrose-associated place preference and may be a viable option to disrupt expression of maladaptive sucrose-seeking behaviors.

Oxytocin's effects on reward-seeking behavior for natural and drug reward have been previously documented, with oxytocin reducing alcohol- (King and Becker, 2019), cocaine- (Zhou et al., 2015b; Leong et al., 2016; Kohtz et al., 2018; Weber et al., 2018), opioid- (Kovács et al., 1985; Sarnyai and Kovács, 1994; Zanos et al., 2014), and methamphetamine- (Carson et al., 2010; Cox et al., 2013, 2017; Everett et al., 2020) seeking behavior, alcohol (Peters et al., 2017) and sugar consumption (Zhou et al., 2015a), craving for marijuana in marijuana-dependent individuals (McRae-Clark et al., 2013), and opioid craving in opioid-dependent individuals (Moeini et al., 2019). Although shown using different paradigms, there is abundant evidence for oxytocin's ability to attenuate drug-seeking behaviors. The present study adds to this body of literature suggesting that oxytocin is also capable of inhibiting the expression of reward-context associations once these associations have been established.

Oxytocin's effects on reward-context associations are likely acting through its well-established interactions with various structures within the reward circuit. The medial pre-frontal cortex (mPFC) has been implicated in the contextual aspects of reward (Haber and Knutson, 2010), possibly through its projections to the dorsal hippocampus (Le Merre et al., 2018). The mPFC also contains reciprocal glutamatergic (afferent) and dopaminergic (efferent) projections with the ventral tegmental area (VTA; Qi et al., 2009). Oxytocin exerts inhibitory effects within the mPFC via the release of γ-aminobutyric acid (GABA) from interneurons onto glutamatergic neurons (Qi et al., 2012). Another study also indicated a potential inhibitory role of oxytocin in the mPFC, as peripheral oxytocin attenuated cocaine cue-induced Fos expression (Leong et al., 2017). This oxytocin-mediated inhibitory response within the mPFC has been shown to disrupt stress-induced reinstatement of methamphetamine CPP (Qi et al., 2009). The amygdala has previously been shown to be involved in the consolidation and expression of amphetamine-induced conditioned place preference (Hiroi and White, 1991; Hsu et al., 2002). Furthermore the central amygdala (CeA), specifically, has been implicated in the acquisition (Rezayof et al., 2007; Li et al., 2011) and expression (Li et al., 2011) of morphine CPP. Oxytocin has been found to excite GABAergic interneurons within the CeA to attenuate emotional responses (Huber et al., 2005; Knobloch et al., 2012). It stands to reason that this inhibitory response from oxytocin might disrupt reward-associated conditioned place preference. There are also a number of studies that suggest oxytocin exerts influence directly within the nucleus accumbens (NAc) to modulate reward-seeking behaviors. Previous research has shown that infusion of oxytocin directly into the NAc attenuates methamphetamine-seeking behavior (Baracz et al., 2016; Bernheim et al., 2017; Cox et al., 2017). Furthermore, peripheral administration of oxytocin has been found to normalize cued cocaine-induced Fos expression in the NAc, demonstrating a potential normalizing effect of oxytocin (Leong et al., 2017). Oxytocin has also been shown to affect extracellular dopamine levels in the NAc after being injected into the VTA (Melis et al., 2007).

Previous studies have shown that oxytocin disrupted the acquisition of methamphetamine (Baracz et al., 2012) and oxycodone (Fan et al., 2019) conditioned place preference when administered prior to conditioning sessions. The results of the present study show no effect of oxytocin administration during conditioning in acquisition of sucrose conditioned place preference. These differences might be due to natural and drug reward being mediated by overlapping yet distinct neural pathways (Nestler, 2005; Alhadeff et al., 2019), resulting in a differential effect of oxytocin at various stages of reward-seeking behaviors. Furthermore, it is also likely that, unlike drugs of abuse, the relatively lower hedonic and incentive value of sucrose is insufficient for oxytocin to exert an effect during acquisition (Kelley and Berridge, 2002). Interestingly, oxytocin has also been shown to enhance the expression of morphine-induced conditioned place preference, but not acquisition, when injected intracerebroventricularly (Moaddab et al., 2015). While these results may directly conflict with the present findings and findings of previous studies showing an attenuating effect of oxytocin on drug-seeking behavior, it is possible that oxytocin facilitates expression of morphine CPP due to morphine's effects on endogenous oxytocin signaling (Kovács et al., 1987). Previous studies have shown that expression of oxytocin receptor and μ-opioid receptor in regions such as the central amygdala (CeA) may result in oxytocin-mediated modulation of morphine's effect (Han and Yu, 2009). Additionally, oxytocin has been shown to act as a positive allosteric modulator of μ-opioid receptors (Meguro et al., 2018), which might explain the facilitating effect of oxytocin on morphine CPP but not for other drugs of abuse or natural rewards.

Some studies have questioned the ability for peripherally-administered oxytocin to cross the blood-brain barrier (BBB) (Ermisch et al., 1985; Kang and Park, 2000), although recent studies have suggested otherwise (Neumann et al., 2013). Peripheral administration of oxytocin caused a rapid increase of oxytocin microdialysates measured in the dorsal hippocampus and amygdala (Neumann et al., 2013). In addition, a central administration of an oxytocin receptor antagonist inhibited the effects of peripherally injected oxytocin on heroin self-administration, morphine tolerance, and cocaine seeking (Sarnyai et al., 1991; Sarnyai and Kovács, 1994). These results suggest that peripheral oxytocin drives behavioral effects through a central mechanism, either by passage through the BBB or through a feed-forward central-release mechanism. For example, peripheral oxytocin suppresses meth-seeking behavior by potentially drive central oxytocin signaling via vagus nerve projections (Everett et al., 2020). Regardless, future studies should examine the effect of centrally-infused oxytocin on sucrose conditioned place preference. Oxytocin has been shown to reduce sucrose and food intake in rats (Zhou et al., 2015a). Both intraperitoneal and intracerebroventricular injection of oxytocin dose-dependently decreased food intake in rats with *ad libitum* food and food-restricted rats (Arletti et al., 1989, 1990). Similarly, central oxytocin injections have been shown to decrease intake of a sucrose solution (VTA: Mullis et al., 2013; NAc core but not shell: Herisson et al., 2016). Intravenous oxytocin has also been shown to reduce food intake in food-deprived rats, but showed no effect on intake of a sugar solution (Klockars et al., 2017). While it is possible that oxytocin's anorexic effect may have influenced the ability of oxytocin to attenuate sucrose place preference, particularly when administered during conditioning, we found no reduction in sucrose pellet intake following oxytocin administration in sucrose-paired conditioning sessions. Furthermore, the results and experimental paradigm of our study examine the effect of oxytocin on sucrose-seeking behavior as there is no sucrose present during testing. Therefore, while oxytocin has previously shown to influence food/sucrose-taking behavior, the present study complements this literature by showing oxytocin also influences sucrose-seeking behavior.

Our present findings demonstrate that oxytocin successfully disrupts sucrose conditioned place preference only after conditioning has been successfully established but does not impact the acquisition of conditioned place preference. Several factors might be driving the oxytocin-specific effect on sucrose place preference expression. First, previous studies have demonstrated that the hippocampus is a key structure facilitating the formation and expression of conditioned place preference (Rezayof et al., 2003). In particular, dorsal hippocampus CA1 D2 receptors have been implicated in the expression of drug-associated conditioned place preference (Haghparast et al., 2013; but see Maldonado et al., 1997). A number of studies have suggested that oxytocin interacts with D2 signaling within the hippocampus (Lazzari et al., 2019). Oxytocin receptors are located on GABAergic interneurons within the dorsal hippocampus (Zaninetti and Raggenbass, 2000). These GABAergic interneurons mediate D2-mediated signaling within the hippocampus (Yoon et al., 2015) and it stands to reason that oxytocin might exert its effect on D2 receptor signaling, and thus expression of place preference, via this circuit. Alternatively, the opioid system has been implicated in the expression, but not acquisition, of conditioned place preference of natural reinforcers (Mehrara and Baum, 1990). The opioid component of neural reward circuits is further supported in studies demonstrating that the μ-opioid receptor agonist, buprenorphine, attenuates expression of cocaine conditioned place preference (Kosten et al., 1991; Suzuki et al., 1992). A number of studies have investigated oxytocin's interactions with the μ-opioid receptor. For example, oxytocin-induced nociception is blocked by intra-NAc infusion of μ-opioid receptor antagonist (Gu and Yu, 2007). Therefore, oxytocin's effect on expression of conditioned place preference could also be driven by its interaction with the endogenous opioid system.

Previous studies have shown that oxytocin produces sex-specific differences in natural and drug related reward-seeking behaviors (Cox et al., 2013; Zhou et al., 2015a; Leong et al., 2016) and that oxytocin receptor expression in the brain show sex-specific differences (Dumais and Veenema, 2016). These studies highlight that the oxytocin system and the behaviors it modulates may be sexually dimorphic and that efforts should be made to directly compare effects of oxytocin between males and females. While the present study investigated the effect of oxytocin on sucrose-seeking behavior in male rats, we used a dose of oxytocin (1 mg/kg) that has been shown to effectively reduce sucrose reinstatement in males and females (Zhou et al., 2015a). However, future studies should examine the effect of oxytocin in sucrose conditioned place preference in females as well.

In conclusion, the present study demonstrates that oxytocin can sufficiently reduce expression of sucrose-mediated conditioned place preference. Further studies should be carried out to determine the specific structures and mechanisms involved in this process. The results presented here provide clinical significance, given the health risks associated with excessive sugar consumption. Furthermore, these results provide additional insight into the processes that underlie oxytocin's effect on reward-related maladaptive behaviors.

## Data Availability Statement

The raw data supporting the conclusions of this article will be made available by the authors, without undue reservation.

## Ethics Statement

The animal study was reviewed and approved by Institutional Animal Care and Use Committee (IACUC) of Trinity University.

## Author Contributions

K-CL, DP, MS, and EL conceived, designed, and performed the experiments. K-CL and DP analyzed the data. K-CL, DP, MS, and EL wrote and edited the manuscript. All authors contributed to the article and approved the submitted version.

## Conflict of Interest

The authors declare that the research was conducted in the absence of any commercial or financial relationships that could be construed as a potential conflict of interest.
